# Metagenomic analysis of human-biting cat fleas in urban northeastern United States of America reveals an emerging zoonotic pathogen

**DOI:** 10.1038/s41598-020-72956-x

**Published:** 2020-09-24

**Authors:** Francisco C. Ferreira, Dina M. Fonseca, George Hamilton, Dana Price

**Affiliations:** 1 Center for Vector Biology, Department of Entomology, Rutgers University, New Brunswick, NJ 08901 USA; 2 Smithsonian Conservation Biology Institute, Washington, DC 20008 USA

**Keywords:** Computational biology and bioinformatics, Genetics, Microbiology

## Abstract

An infestation of cat fleas in a research center led to the detection of two genotypes of *Ctenocephalides felis* biting humans in New Jersey, USA. The rarer flea genotype had an 83% incidence of *Rickettsia asembonensis*, a recently described bacterium closely related to *R. felis,* a known human pathogen. A metagenomics analysis developed in under a week recovered the entire *R. asembonensis* genome at high coverage and matched it to identical or almost identical (> 99% similarity) strains reported worldwide. Our study exposes the potential of cat fleas as vectors of human pathogens in crowded northeastern U.S, cities and suburbs where free-ranging cats are abundant. Furthermore, it demonstrates the power of metagenomics to glean large amounts of comparative data regarding both emerging vectors and their pathogens.

## Introduction

Numbers of reported cases of diseases transmitted by mosquitoes, ticks, fleas and other vectors (vector-borne diseases), tripled across the U.S. between 2004 and 2016^1^ and the trend is continuing even with rampant under-reporting^2^. The alphaproteobacterial genus *Rickettsia* is divided into four groups, three of which include vector-borne species pathogenic to humans. These three groups, followed by a representative vector-borne species are: the spotted fever group (SFG), *Rickettsia rickettsii*; the typhus group (TG), *Rickettsia typhi* and the transitional group (TRG), *Rickettsia felis*^3^. Species from all three groups are common in wild and domestic mammals globally; SFG transmission to humans occurs via tick bite, while many arthropods including fleas are known vectors of TG and TRG^4^. Since 2000, cases of presumptive SFG have increased markedly in the mid-Atlantic states, including New Jersey, associated with reduced morbidity and mortality^5^, a puzzling phenomenon since recent surveys of the primary tick vectors have seldomly detected the presence of SFG *Rickettsia*^6^.

Cat fleas (*Ctenocephalides felis*) are the most common ectoparasites of domestic cats and dogs worldwide^7^, can parasitize humans in many areas of the globe^7,8^ and are commonly infected with potentially dangerous pathogens including *Rickettsia* species^9,10^. Studies of flea-borne pathogens commonly focus on surveillance using fleas collected from companion animals or wildlife without examining their potential to feed on humans, thus limiting estimates of risk. To our knowledge, no previous studies have reported cat flea outbreaks affecting humans in the highly urbanized northeastern U.S., although cat flea transmission of endemic typhus (*R. typhi*) and flea-borne spotted fever (*R. felis*) have been reported in southern California and Texas^11^. Overall, human exposure risk to cat flea-borne pathogens in the northeastern U.S. is broadly unknown and potentially underestimated.

Here we report a cat flea infestation at Rutgers University, New Jersey U.S. that eventually included an entomology laboratory at the Center for Vector Biology, NJ Agricultural Experiment Station.

## Results

### Cat flea infestation

**Figure 1:**
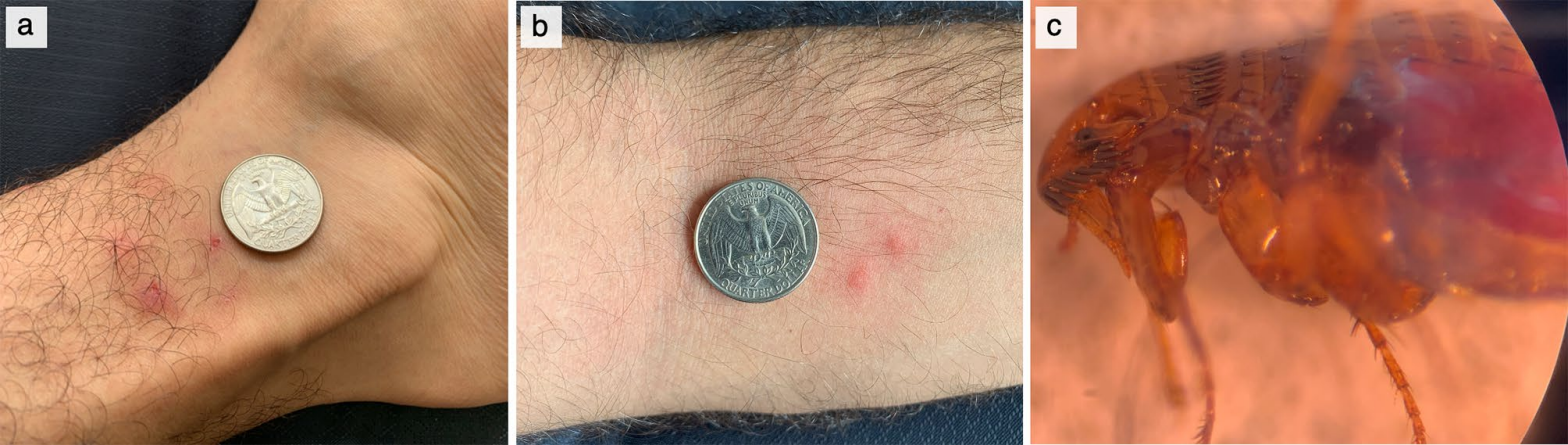
Flea bites and cat flea (*Ctenocephalides felis*) engorged with blood. Flea bites were concentrated on the ankles and front of the arms but bites on the torso and abdomen also occurred. Examples of lesions on the researchers’ ankles (**a)** and anterior arms (**b**). Bite lesions were itchy for over a week and scratching resulted in significant damage to the skin (**a**). A cat flea collected while feeding (**c**).

On July 18, 2019 a Rutgers University maintenance worker discovered large numbers of small insects on his clothing and skin after exiting a nearby work site and rushed to the Center for Vector Biology seeking assistance. The following day, while doing research in one of the center’s laboratories, the first author (FCF) sustained multiple bites with subsequent lesions on legs (Fig. 1a), arms (Fig. 1b) and body, detected the first fleas in one of the center’s laboratories and collected five fleas actively feeding on himself. Subsequently, a large infestation of *Ctenocephalides* sp. fleas was identified at the (index) work site associated with the presence of free-ranging cats, and a secondary infestation of adult fleas (introduced with the maintenance worker) at the research center. The research center was evacuated and extensive meetings among entomologists, facilities, janitorial, occupational health and safety and contract pest exterminator personnel were required to develop a control plan. To limit impact on live insect colonies maintained at the research center but outside the infested quarters, all surfaces in infested areas were treated twice with a short-residual pyrethrin followed each time by extensive washing and vacuuming of floors and carpets. To assess control efficacy, the exterminators deployed glue boards in every room and light traps in the corridors. The infestation was declared over after 2 weeks when no new fleas were captured for three consecutive days.

In addition to the five fleas that were collected feeding, we obtained 102 fleas from glue traps deployed in both infested sites, which were all morphologically identified as cat fleas (*Ctenocephalides felis,* Fig. 1c) based on three diagnostic characters: slightly convex shape of the head; first spine of the genal ctenidia exceeding at least half of the second in length; and presence of six spiniform setae in the dorsoposterior margin of the hind tibia. We extracted DNA individually from all 107 fleas and selected a sub-sample of 37 for molecular barcoding at the cytochrome c oxidase subunit I (*coxI*) locus. This revealed two *coxI* haplotypes of *C. felis* that matched those from a recent worldwide assessment of genetic diversity in *Ctenocephalides* fleas^7^. We found 31 fleas with *coxI* haplotype h1 (Accession no. MT901293) and 6 fleas with *coxI* haplotype h6, Accession no. MT901294), which differs from h1 by 2.1% at the nucleotide level. Haplotype h1 is distributed across the temperate zone in all continents except Antarctica and was also recently found in a Long Island, NY (cat flea sequenced as part of bioproject PRJNA184075, Accession no. MK481256). Haplotype h6 is known to occur in the Central African Republic and the Seychelles^7^ and has been detected in Georgia, USA^12^. Four of the five fleas collected while feeding on humans had the h1 haplotype and one had the h6 haplotype. Fleas with different haplotypes were morphologically similar.

Although the bites experienced indicate that fleas were biting humans, we amplified and sequenced a 741 bp fragment of vertebrate *cytb* locus (Ngo & Kramer 2003) from a h6 haplotype flea with a visible blood meal to confirm that they were successfully obtaining human blood and not just probing. We obtained a clean sequence of *Homo sapiens cytb* (Accession no. MT901295) confirming the blood in the flea was from a human.

### *Rickettsia* detection in cat fleas

We tested all 107 fleas for *Rickettsia* sp. in pools of DNA with up to eight specimens using a qPCR assay targeting the conserved 17kD antigen locus^13^. Fleas from six positive pools were individually tested with the same assay, revealing six positive individuals (5.6%) with a single positive per pool. We then amplified and sequenced fragments of the *gltA*^6^ (Accession no. MT901296) and *ompB*^14^ (Accession no. MT901297) genes from all six positive fleas, which generated a single genotype for each gene. These sequences produced a 100% match with isolates previously described as *Rickettsia asembonensis* in California, US^15^, Peru^16^ and Brazil^17^. We found that five of the six infected fleas had haplotype h6 (83% of h6 fleas were positive for *R. asembonensis*) and one had haplotype h1 (3% of h1 fleas were positive for *R. asembonensis*). These infection rates are significantly different (Fisher's Exact Test; *P* < 0.001).

### Metagenomic analyses: *Rickettsia asembonensis* genome and cat flea mitochondrial genome

We selected a h6 *Rickettsia*-positive cat flea that was trapped in a glue trap for a metagenomic analysis using next-generation Illumina sequencing and generated 12.19 M paired-end reads (24.38 M total reads) of 305 bp that assembled to 399,287 scaffolds with an N50 of 1.4Kbp and summed to 430 Mbp. Of these scaffolds, 391,098 (98.0%) had hits to the database (e-val = 1 × 10^−10^; Table S1); 390,877 (99.94%) were to *C. felis* genome scaffolds. Of the remaining scaffolds, 93 had top hits to *Rickettsia* bacteria and summed to 1.34 Mbp with an average nucleotide coverage of 85×, suggesting that we had a complete (or near-complete) bacterial genome using the 1.37 Mbp published *R. asembonensis* genome isolated from Kenya^18^ and cultured in an *Aedes albopictus* cell line as a reference. Using this published bacterial genome, we identified 2373 single nucleotide variants (SNVs) with > 10 × coverage at 95% frequency in our short-read data, indicating that the two strains were > 99.99% identical on average at the nucleotide level.

We used the Illumina metagenome assembly data to compare the *R. asembonensis* we detected with *R. asembonensis* isolates from around the world by deriving full-length gene sequences for five commonly sequenced rickettsial marker genes, which include conserved (*gltA* and 17-kDa) and variable (*ompB*, *ompA* and *sca4*) loci. Gene sequences identical or almost identical (> 99.9% identity) to those from our study were detected in cat fleas from California, Texas and Georgia within the U.S. and in cat fleas and other vectors in countries in Central and South America, Europe, Africa and in Asia (Table 1). The *R. asembonensis* detected here is similar (> 99%) to genotypes obtained from human blood in Peru^19^ and Malaysia^20^, from Long-tailed macaques (*Macaca fascicularis*) in Malaysia^21^ and from cats in Thailand^22^.

**Table 1:** Gene comparison between the *Rickettsia asembonensis* complete genome from this study and those previously detected in fleas, ticks and in blood samples across the world.

Source	Vertebrate host	Location, Country	Year^1^	*gltA*		17 kDa		*ompB*		ompA		Reference
				Identities^2^	GenBank acc. no	Identities^2^	GenBank acc. no	Identities^2^	GenBank acc. no	Identities^2^	GenBank acc. no	
*Ctenocephalides felis*	Human (*Homo sapiens)* Grizzly (*Ursus arctosi*)	Columbia, SC, USA	2009	371/372 (99.7%)	AY953289	432/434 (99.5%)	AY953286					Nelder et al.^40^
*C. felis*	Opossum (*Didelphis virginiana*)	Orange Co., CA, USA	2016	**1035/1035 (100%)**	KU597068			**1517/1517 (100%)**	KU597066	**1224/1225 (99.9%)**	KU597069	Krueger et al.^15^
*C. felis*	Cat (*Felis catus*)	Los Angeles Co., CA, USA	2016					**787/787 (100%)**	KP398499			Billeter et al.^41^
*C. felis*	Cat (*Felis catus*)	Georgia, USA	2016	**613/613 (100%)**	KX431974					**833/833 (100%)**	KX431985	Šlapeta and Šlapeta^12^
*C. felis*	Opossum (*Didelphis virginiana*)	Orange Co., CA, USA	2016					**787/787 (100%)**	KP398499^4^			Maina et al.^29^
*C. felis*	Cat (*Felis catus*)	Galveston, TX, USA	2019	**342/342 (100%)**	MH325379	**394/394 (100%)**	MH325383	**769/770 (99.9%)**	MH325368			Blanton et al.^4^
*C. felis*	Dog (*Canis lupus*)	Coclé province, Panama	2011	**724/724 (100%)**	HM582437							Bermudez et al.^42^
*Pulex simulans*	Dog (*Canis lupus*)	Costa Rica	2016	**350/350 (100%)**	KJ569090^5^							Troyo et al.^43^
*Amblyomma ovale*	Dog (*Canis lupus*)	Costa Rica	2016	**349/349 (100%)**	KX544811			**559/559 (100%)**	KX544817			Troyo et al.^43^
*Rhipicephalus* *microplus*	Cow (*Bos taurus*)	Costa Rica	2016	**350/350 (100%)**	KJ569090^5^							Troyo et al.^43^
*C. felis*	Collected from a bed	Villeta, Colombia	2016	**350/350 (100%)**	KJ569090			**382/382 (100%)**	KJ569091			Faccini-Martínez et al.^44^
*C. felis*	Dog (*Canis lupus*)	Imperatriz, Brazil	2017	**1018/1018 (100%)**	KY445726	**528/528 (100%)**	KY445730	**794/794 (100%)**	KY445737			Silva et al.^17^
*C. felis*	Dog (*Canis lupus*)	Edison Lobão, Brazil	2017	**1109/1110 (99.9%)**	KY445725	**532/532 (100%)**	KY445735	**762/762 (100%)**	KY445740			Silva et al.^17^
*Rhipicephalus sanguineus*	Dog (*Canis lupus*)	Tapes, Brazil	2017	**1136/1137 (99.9%)**	KX196267	**369/369 (100%)**	KX196268					Dallagnol et al.^45^
*C. felis* VGD7	NA	Iquitos, Peru	2018	**1314/1314 (100%)**	KY650697	**476/476 (100%)**	KY650697	**4944/4947 (99.9%)**	KY650699	**4159/4164 (99.9%)**	KY650698	Loyola et al.^16^
*Ctenocephalides canis* 8294D3 *C. felis LER205*	NA NA	Iquitos, Peru Puerto Maldonado, Peru	2019	**1314/1314 (100%)** **1314/1314 (100%)**	MK923743 MK923726	**476/476 (100%)** **476/476 (100%)**	MK923744 MK923729	**4947/4947 (100%)** **4947/4947 (100%)**	MK923741 MK923726	**5151/5151 (100%)** **5151/5151(100%)**	MK923742 MK923727	Loyola et al.^16^
Blood	Human (*Homo sapiens*)	Various localities, Peru	2018	382/383 (99.7)	LN831076							Palacios-Salvatierra et al.^19^
*Archaeopsylla erinacei*	Hedgehog (*Erinaceus europaeus*)	Munich, Germany	2009	**335/335 (100%)**	EU927696			**770/770 (100%)**	EU927697			Gilles et al.^46^
*Pulex irritans*	Dog (*Canis lupus*)	Hungary	2010	**341/341 (100%)**	EU853838							Hornok et al.^47^
*Echidnophaga gallinacea*	Black rats *Rattus rattus)*	Egypt	2006	**341/341 (100%)**	DQ166938	**394/394 (100%)**	DQ166937					Loftis et al.^48^
*Synosternus pallidus*	Cat (*Felis catus*), Dog (*Canis lupus*, traps in homes	Sine-Saloum, Senegal	2012	726/728 (99.7%)	JF966774			**770/770 (100%)**	JF966775			Roucher et al.^49^
*C. canis*	Dog (*Canis lupus*)	Nyanza, Kenya	2013	1127/1130 (99.7%)	JN315968	**371/371 (100%)**	JN315968	819/820 (99.8%)	JN315972	**1515/1517 (99.9%)**	JN315977	Jiang et al.^50^
*C. felis*	Dog (*Canis lupus*)	Nyanza, Kenya	2013	1311/1314 (99.8%)	JWSW01000078	**501/501** **(100%)**	JWSW01000044	**4944/4947** **(99.9%)**	JWSW01000087	5080/5088(99.8%)	JWSW01000076	Jima et al.^18^
*C. felis*	Dog (*Canis lupus*)	Musanze, Rwanda	2018	611/612 (99.8%)	MH142453					831/833 (99.7%)	MH142452	Nziza eet al.^51^
*C. felis*	Dog (*Canis lupus*)	Mazabuka, Zambia	2019	320/321 (99.7%)	LC431490			**696/696 (100%)**	LC431491	**208/208 (100%)**	LC431502	Moonga et al.^52^
*C. canis*	Dog (*Canis lupus*)	Sangkhlaburi, Thailand	2003	1166/1171 (99.6%)	AF516333			772/774 (99.7%)	JX183538			Parola et al.^53^
*C. felis*	Dog (*Canis lupus*)	Bangkok, Thailand	2011	**328/328 (100%)**	JF511463	**390/390 (100%)**	JF511461					Foongladda et al.^54^
*C. felis*	Dog (*Canis lupus*)	Bangkok, Thailand	2018	132/134 (98.5%)	MG264737	304/305 (99.7)	MG452137					Mongkol et al.^55^
*C. felis*	Dog (*Canis lupus*)	Nan province, Thailand	2019	**525/525 (100%)**	MK660561							Takhampunya et al.^56^
Blood	Cat (*Felis catus*)	Bangkok, Thailand	2018	1160/1168 (99.3%)	MH523411							Phoosangwalthong et al.^22^
*C. felis*	Dog (*Canis lupus*) Cat (*Felis catus*)	Kuala Lumpur, Malaysia Pulau Pinang, Malaysia	2014	**725/725 (100%)**	KF963606							Tay et al.^57^
*Ctenocephalides orientis*	Dog (*Canis lupus*)	Peninsular Malaysia	2017	**1136/1137 (99.9%)**	KX196267^3^							Low et al.^58^
*Rhipicephalus sanguineus*	Dog (*Canis lupus*)	Peninsular Malaysia	2017	**748/749 (99.9%)**	MF281711							Low et al.^58^
Blood	Long-tailed macaque (*Macaca fascicularis*)	Peninsular Malaysia	2015	**210/210 (100%)**	KP126803			777/779 (99.7%)	KP126804			Tay et al.^21,22^
Blood	Human (*H. sapiens*)	Kuala Lumpur, Malaysia	2016	399/402 (99.2%)	KU255716			816/818 (99.8%)	KU255717			Kho et al.^20^
*Ceratophyllus fasciatus*	Rat (Rattus rattus)	Himachal Pradesh, India	2015	**341/341 (100%)**	HM370112			792/794 (99.7%)	HM370113			Cahota et al.^59^
*C. orientis*	Dog (*Canis lupus*)	Delhi, Mumbai, Rajasthan, India	2015	**586/586 (100%)**	KP256357					**794/795 (99.9%)**	KP256359	Hii et al.^60^
*Xenopsylla ramesis*	Wild rodents	Negev, Israel	2015	**340/340 (100%)**	KP050777	**332/332 (100%)**	KP050778	**739/740 (99.9%)**	KP050780	**505/505 (100%)**	KP050779	Rzotkiewicz et al.^61^

Identical and almost identical (> 99.9%) matches are in bold.

The *sca4* gene was omitted from the table to reduce its size. This gene was sequenced in only 4 studies^15,16,18,50,61^, which produced sequences with 100% identity in relation to the *R. asembonensis* detected in this study.

NA = Information not available, *C. felis* = *Ctenocephalides felis*, *C. canis* = *Ctenocephalides canis*, *C. orientis* = *Ctenocephalides orientis.*

^1^Refers to the year of publication.

^2^ refers to the number of identical bp in the comparison, the total number of overlapping bp and the similarity percentage (in parenthesis).

^3^While Low et al. 2017 did not create a GenBank entry for their *gltA* sequences they stated the genotype they found was a 100% match to acc. number KX196267.

^4^While Maina et al. 2016 did not create a GenBank entry for their *ompB* sequences they stated the *ompB* genotype they found was a 100% match to acc. number KP398499.

^5^While Troyo et al. 2016 did not create a GenBank entry for some of their *gltA* sequences they stated that the genotype they found was a 100% match to the acc. number KJ569090.

**Figure 2:**
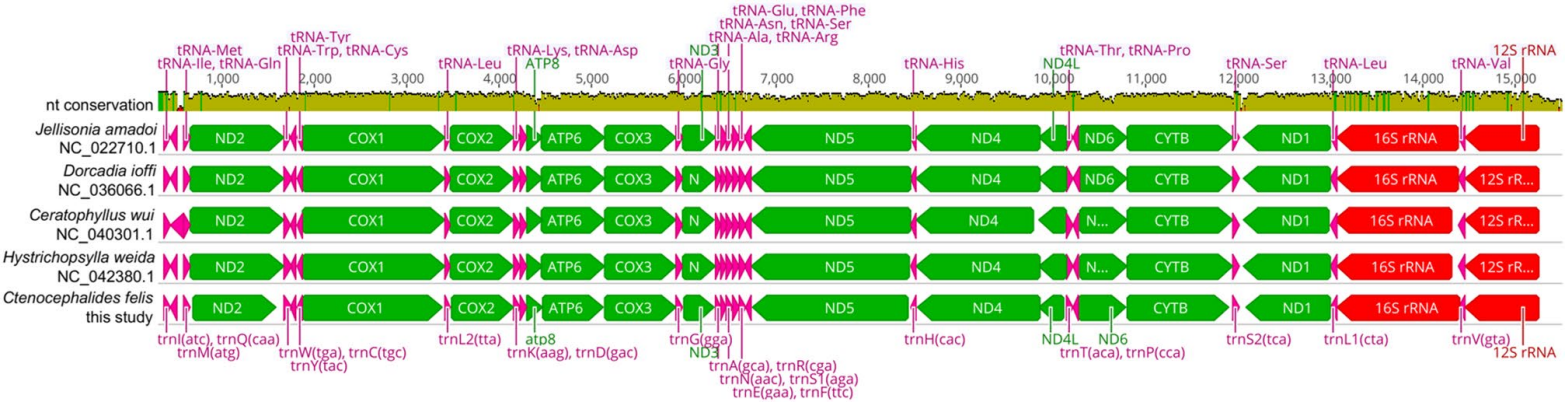
Mitochondrial synteny diagram illustrating gene order and nucleotide sequence conservation between *Ctenocephalides felis* (generated in this study) and four currently sequenced flea mitochondrial genomes. The control region is excluded.

In addition to the *R. asembonensis* genome, we identified a scaffold encoding a near full-length *C. felis* mitochondrial genome. This 17.54 kbp scaffold (NODE_28 within the assembly) encodes 13 protein coding genes, 22 tRNAs and two rRNAs and had a mean mapping coverage of > 526×. We were unable to circularize the genome due to a high number of tandem repeats in the control region that could not be spanned by short-read sequencing. Annotation and alignment with four existing Siphonapteran mitogenomes currently available^23–25^ (Fig. 2) indicate that gene order and sequence within the coding region remain relatively conserved among the three infraorders (Ceratophyllomorpha, Pulicomorpha, Hystrichopsyllomorpha) and four families sampled (Ceratophyllidae, Vermipsyllidae, Hystrichopsyllidae, Pulicidae).

## Discussion

Our unanticipated study uncovered two different mitochondrial lineages of human-biting cat fleas infected with *Rickettsia asembonensis*. This flea-borne spotted fever *Rickettsia* species falls within the transitional group^26^ and has been associated with human pathogenicity in Peru^19^ and in Malaysia^20^. *Rickettsia asembonensis* was detected in the blood of healthy cynomolgus monkeys (*Macaca fascicularis*) in Malaysia^21^ and in blood samples from cats in Thailand^22^, showing that this bacterium infects wild and domestic mammals, which in turn can act as reservoirs for human infections. To date, *R. asembonensis* has been detected in three tick species and in 10 flea species from 17 countries across four continents; the global distribution of positive cat fleas suggest they are likely to be vectors of this *Rickettsia* species. More importantly, regions with confirmed cases of *R. asembonensis* infection in humans and other mammals also have reports of positive cat fleas (Table 1). The cat flea is the only known vector of *R. felis*^27^, and currently stands as the main vector of typhus fever in the U.S.^4,28^, reinforcing its importance as a vector of zoonotic pathogens.

We found that fleas with both h1 and h6 *coxI* haplotypes bit humans and we identified human blood in a haplotype h6 flea infected with *R. asembonensis*. Of note, although the dozens of skin lesions resulted in a few days of significant discomfort no further symptoms have surfaced*.* While flea-borne rickettsioses in humans have been linked to the presence of *Rickettsia-*positive cat fleas^29^, direct evidence of positive fleas biting humans is still scarce. The significant difference in infection rate between fleas with different *coxI* haplotypes raises questions about differences in vector competence or likelihood of vertical transmission of *R. asembonensis* in different genetic lineages of cat fleas*.*

In the U.S., human cases of flea-borne diseases have been reported in Hawaii, Texas and California^30^ and our study brings attention to the possible emergence of flea-borne pathogens in the northeastern region where millions of cats and dogs thrive alongside humans^31^. In fact, the cat flea infestation we report led to the discovery of a medium sized colony of free-ranging cats in the basement of the primary infestation site. Our results warrant increasing the public’s awareness that free-ranging cats can harbor fleas infected with zoonotic pathogens. Of note, serological assays for other rickettsial agents such as *Rickettsia typhi* (murine typhus) and *R. rickettsii* (the agent of Rocky Mountain spotted fever) cross-react with *R. asembonensis*^32^ and the latter should therefore be considered when diagnosing fevers of unknown origin in the northeastern U.S.

The ability to sequence and assemble an entire rickettsial genome from a single infected flea illustrates the potential that modern metagenomic analyses hold for vector biology and public health. We demonstrated that the *R. asembonensis* detected here is highly similar to those distributed in many countries across the Americas, Europe, Africa and Asia. These results are currently tempered, however, by a lack of comparative data in public databases e.g., GenBank, with a large majority of data from closely related *Rickettsia* species existing as single genes, partial gene fragments, or missing entirely. Recovery of the complete mitochondrial genome of *C. felis* concomitant with the pathogen genome enables expanded genotype by genotype evolutionary analyses and provides data useful for population-level analyses using mitochondrial markers. We envision that as metagenome sequencing becomes cheaper and commonplace in situations such as this, comparative analyses within and between global vector populations and their pathogens (inclusive of bacteria, viruses and eukaryote parasites) will be possible, and will quickly expand our knowledge about potential emerging pathogens to which human populations are exposed.

## Methods

### Flea sampling, identification and DNA extraction

One of the authors of this study (FCF) caught five fleas that were feeding on himself and put these specimens in microtubes containing 95% ethanol. We also obtained fleas that were trapped in glue boards (Catchmaster Mouse and Insects glue boards, Catchmaster, NJ, US) and light traps (Victor Ultimate Flea Trap, Woodstream Corporation, PA, US) deployed in the halls and offices of the infested buildings for 2 weeks. We inspected the traps daily, transferred glue boards positive for fleas to a − 20 °C freezer and later removed trapped fleas with forceps before extraction.

Fleas were identified to the species level with the aid of a taxonomic key (Centers for Disease Control and Prevention^33^). We then extracted DNA from all 107 fleas individually using a phenol–chloroform protocol^34^.

### Genetic identification of fleas and blood meal analysis

To confirm the flea species we used primers LCO1490 and ﻿Cff-R^35^ to amplify and sequence the mitochondrial cytochrome oxidase 1 (*coxI*) barcode locus from 37 fleas (six *Rickettsia*-positive fleas), which included one collected feeding on a human (FCF), four other fleas collected on this same person and 27 additional fleas chosen randomly from those available. We amplified and sequenced a 741 bp fragment of vertebrate *cytb* locus using primers MammalianF and MammalianR^36^ from a flea with a visible blood meal.

### *Rickettsia* detection

We tested all 107 fleas for *Rickettsia* sp. in pools of up to eight specimens using a qPCR assay targeting the conserved 17kD locus^13^. Fleas from positive pools were individually tested with the same qPCR assay to pinpoint which fleas in each pool were positive. To identify the *Rickettsia* to species level we amplified and sequenced a 608 bp fragment of the rickettsia *gltA* gene using primers PgltA-2F 5′-TTCTCATCCTATGGCTATTATGC-3′ and PgltA-2R 5′-TTCAAGTTCTATTGCTATTTG-3′^6^ and a 820 bp fragment of the rickettsia *ompB* gene using primers 120-M59 and 120–807^14^. Positive and negative controls produced the expected results in all tests performed.

### Metagenomic analysis

We used approximately 50 ng of DNA from an un-engorged *Rickettsia*-positive flea (haplotype h6) to construct an Illumina shotgun sequencing library using the Nextera FLEX sample prep kit (Illumina Inc, San Diego, CA) and a 600-cycle version 3 sequencing kit in 305 bp × 305 bp paired-end mode. Raw reads were adapter and quality-trimmed using BBduk (sourceforge.net/projects/bbmap/) and assembled using SPAdes 3.14.0^37^. Each scaffold was queried against the NCBI ‘nt’ nucleotide database with the addition of the *C. felis* draft genome sequence (NCBI accession GCF_003426905; Driscoll et al. [unpublished]) using BLASTN 2.9.0+^38^ on the Rutgers amarel high-performance computing cluster, and hits were annotated with corresponding NCBI taxonomy using the Taxonomizr R module (https://cran.r-project.org/web/packages/taxonomizr/index.html). Using BLAST homology to *Rickettsia* CDS sequences within NCBI, we identified full-length homologs of the *gltA*, *ompB*, *ompA*, *sca4* and the 17 kDa outer membrane antigen *htrA* within this assembly. Raw reads were additionally mapped to the *R. asembonensis* strain NMRCii genome scaffolds and plasmid pRAS01^18^ [GenBank accession GCA_000828125.2]) using BBmap. Reads with > 1 best alignment were discarded. Of the 23.24 M trimmed reads (totaling 4.51Gbp), 600 k (2.58%) were mapped to the reference. We used this mapping with the Geneious Prime v.2020.1.2 variant caller (Biomatters, Ltd., Auckland, NZ) to assess the number of single-nucleotide variants (SNVs) between the two *R. asembonensis* genomes. The scaffold encoding the *C. felis* mitochondrial genome was annotated using MITOS^39^.

## Ethical statements

All methods were carried out in accordance with relevant guidelines and regulations. The research reported does not involve experiments on humans or animals and therefore neither IRB nor IACUC protocols were necessary. Instead it reports the outcome of an inadvertent, unexpected and undesired ectoparasite infestation in our research center. Fleas were collected by the first author from his own person upon infestation of the research center.

## Supplementary information

Supplementary Legends.

Supplementary Table S1.

Supplementary Table S2.

## Data Availability

The datasets generated during and/or analysed during the current study are available in GenBank under Accession Numbers MT901293–MT901297 for the sequences obtained via Sanger sequencing. The assembled metagenome scaffolds and raw sequence reads are available via NCBI BioProject PRJNA659057.
