# Supplementary material for: Metagenomic analysis of human-biting cat fleas in urban northeastern United States of America reveals an emerging zoonotic pathogen
Source: Sci Rep. 2020 Sep 24;10:15611. doi: 10.1038/s41598-020-72956-x (PMC7519146; doi:10.1038/s41598-020-72956-x)
Supplement: Supplementary file 1 — Supplementary Legends. [file 41598_2020_72956_MOESM1_ESM.docx]

**Metagenomic analysis of human-biting cat fleas in urban northeastern United States of America reveals an emerging zoonotic pathogen**

Francisco C. Ferreira^1,2*^, Dina M. Fonseca^1^, George Hamilton^1^, Dana Price^1^

^1^Center for Vector Biology, Rutgers University, New Brunswick NJ, 08901 USA

^2^Smithsonian Conservation Biology Institute, Washington DC, 20008 USA

*email: [franciscocarlosfj@gmail.com](mailto:franciscocarlosfj@gmail.com)

**Table S1. BLASTn top hit output and taxonomic assignment for metagenome scaffolds.** Hits derived from the *C. felis* genome^17^ and not the NCBI 'nt' database are prefixed with “CFELIS”.

**Table S2. *C. felis* mitochondrial scaffold (NODE_28) annotation table.**
